# In Vitro Miniaturized Tuberculosis Spheroid Model

**DOI:** 10.3390/biomedicines9091209

**Published:** 2021-09-13

**Authors:** Shilpaa Mukundan, Pooja Singh, Aditi Shah, Ranjeet Kumar, Kelly C. O’Neill, Claire L. Carter, David G. Russell, Selvakumar Subbian, Biju Parekkadan

**Affiliations:** 1 Department of Biomedical Engineering, Rutgers, The State University of New Jersey, Jersey City, NJ 08854, USA; fs386@scarletmail.rutgers.edu (S.M.); ars340@scarletmail.rutgers.edu (A.S.); 2 Public Health Research Institute, New Jersey Medical School, Rutgers, The State University of New Jersey, Jersey City, NJ 07103, USA; poojasingh@uabmc.edu (P.S.); rk879@njms.rutgers.edu (R.K.); subbiase@njms.rutgers.edu (S.S.); 3 Department Center for Discovery and Innovation, Hackensack Meridian Health, Neptune, NJ 07110, USA; kelly.oneill@hmh-cdi.org (K.C.O.); claire.carter@hmh-cdi.org (C.L.C.); 4 Department of Microbiology and Immunology, School of Veterinary Medicine, Cornell University, Ithaca, NY 14853, USA; dgr8@cornell.edu; 5 Department of Medicine, Rutgers Biomedical Health Sciences, Rutgers, The State University of New Jersey, Jersey City, NJ 08854, USA

**Keywords:** tuberculosis, lipid characterization, MALDI MSI, in vitro model

## Abstract

Tuberculosis (TB) is a public health concern that impacts 10 million people around the world. Current in vitro models are low throughput and/or lack caseation, which impairs drug effectiveness in humans. Here, we report the generation of THP-1 human monocyte/macrophage spheroids housing mycobacteria (TB spheroids). These TB spheroids have a central core of dead cells co-localized with mycobacteria and are hypoxic. TB spheroids exhibit higher levels of pro-inflammatory factor TNFα and growth factors G-CSF and VEGF when compared to non-infected control. TB spheroids show high levels of lipid deposition, characterized by MALDI mass spectrometry imaging. TB spheroids infected with strains of differential virulence, *Mycobacterium tuberculosis* (Mtb) HN878 and CDC1551 vary in response to Isoniazid and Rifampicin. Finally, we adapt the spheroid model to form peripheral blood mononuclear cells (PBMCs) and lung fibroblasts (NHLF) 3D co-cultures. These results pave the way for the development of new strategies for disease modeling and therapeutic discovery.

## 1. Introduction

There is an unmet need in modeling TB infection for the rapid discovery of new diagnostics and therapeutic agents for the disease. In vivo models such as mouse, guinea pig, rabbit and non-human primate have provided tremendous insight into TB disease progression and drug response. Though clinically relevant, these models are labor-intensive and expensive [1]. In vitro TB assays represent disease states to some extent, by tuning oxygen [2,3], nitric oxide [4] and nutrient sources [5,6] to alter mycobacterial growth. In addition, tremendous progress has been made to engineer mycobacteria with florescent reporters [7,8], metabolic engineering and advanced tools such as clustered regularly interspaced short palindromic repeat (CRISPR) interference (CRISPRi) to alter mycobacterial growth and function [9,10]. The models focusing directly on the mycobacteria have been instrumental in identifying differential sensitivities of mycobacteria to isoniazid (INH), rifampicin (RIF) and metronidazole, but they lack host cells and caseum [11]. Subsequent studies incorporated human macrophages or peripheral blood mononuclear cells (PBMC) to recreate caseation by using hypoxia and oleic acid treatment [12], but they lack 3D structure and diffusion limitations. Three-dimensional aggregates of PBMCs are one of the most common in vitro models for studying granuloma formation [13,14] and they possess cellular heterogeneity and cytokine profiles similar to that of human TB patients [1,15]. While these stand-alone PBMC cultures form loose aggregates, efforts to add extracellular matrix proteins such as collagen has enabled formation of aggregates with a necrotic core [16]. The PBMC-based model has also been shown to capture latency [17] and tuberculosis reactivation via TNF-α inhibition [18]; however, they lack experimental control of aggregation. To tackle this, Puissegur et al. developed a PBMC infection model by coating glycine beads with PPD or live bacteria and allowed immune cells to infiltrate on top of the beads mimicking granuloma formation [19]. The size of the granuloma is thus defined by the size of the glycine beads. Similarly, bioengineering approaches such as electro-spraying have also been adopted to generate engineered micro-encapsulated PBMCs in collagen-alginate matrices [20,21]. Similarly, the integration of microsphere technology with microfluidics also demonstrated utility for the study of Mtb antimicrobial resistance [16]. The adaptation of bioengineering techniques towards TB disease modelling can facilitate easy culture of 3D structures. The additional processing steps and equipment needed to generate microcapsules limits the usage of these models for drug screening and harvesting cells for downstream applications. In addition, PBMC-based models do not account for interaction with lung fibroblast, which play a key role in TB infection.

Recent advancements in organoids provide a promising avenue to model tuberculosis granulomas in the context of lung tissue. Currently, alveolar organoids can be grown to a size of 500 µ(micro)m and can serve as a potential model of TB infection [22]. However, the lack of immune cells in this model is a major limitation. Incorporation of immune cells within organoids has been one of the greatest challenges and has been accomplished via addition of patient specific PBMCs [23,24,25]. Recent studies reported the generation of immune organoids with Naïve B cells co-cultured with stromal cells to model lymph node [26,27]. Despite these advances, this field of incorporating stromal cells and immune cells within organoid matrices is still in its infancy. Alternative cell-line derived models such as organ on chip technologies are gaining importance in tuberculosis modelling, specifically for the ability to engineer air-liquid interfaces using microfluidic technologies [22,28,29]. Layered epithelial-fibroblast cells cultured on collagen gels placed in porous membranes, a simpler alternative model, have also been reported to depict air-liquid interface and stimulate mucous microenvironment [30]. Despite these features, the ease of fabrication, throughput and scaling these models to test anti-TB compounds limit their application.

By integrating the learnings from the TB field with the bioengineering field, 3D spheroid models can be adapted to incorporate mycobacteria with the aim to establish easy and scalable, high throughput 3D models. For instance, Harimato et al. developed 3D spheroid models of engineered *Salmonella typhimurium* and cancer cells and characterized colonization and persistence of bacteria inside the spheroids [31]. Similarly, Bartfield et al. modeled gut-bacterial infection using 3D cell cultures [32]. Over the years, spheroid forming technologies have also been tailored towards medium and high throughput drug testing applications [33,34]. Herein, we applied a simple strategy of 3D cell culture using Aggrewell 400™ plates (Stemcell Technologies, Vancouver, BC, Canada) to generate controlled size spheroids using an optimal cell number seeded per well (referred to as TB spheroid) (Figure S1) to model TB granulomas and extract insight into disease pathogenesis and drug responsiveness. Since our model is a spheroid system, it is anchorage independent and does not require addition of extracellular matrix such as collagen. In addition to this, each well contains 1200 microwells. This enables the formation of large number of spheroids within a single well, surpassing the throughput of current in vitro TB models. Further, the TB spheroid model is amenable to addition of multiple cell types, and we demonstrated this using PBMCs co-cultured with normal human lung fibroblasts. The following sections describe the detailed characterization of TB spheroids to study cell viability, bacterial localization, lipid deposition, cytokine profiling and drug responsiveness. This first-generation model and the results observed in the study will enable us to optimize and develop TB spheroids from pathological tissues from animal and human TB patients in the future.

## 2. Materials and Methods

### 2.1. Host Cell Culture

THP-1 human monocyte cells, Jurkat T cells (ATCC, Manassas, VA, USA) were cultured in RPMI media (Gibco, Grand Island, NY, USA) containing 10% fetal bovine serum (FBS) and 1% penicillin and streptomycin (pen/strep). J774 mouse macrophages (ATCC, Manassas, VA, USA) were cultured in Dulbecco’s MEM (DMEM, Gibco, Grand Island, NY, USA) supplemented with 10% FBS, 1% pen/strep. Normal human lung fibroblasts (NHLFs) were obtained from Lonza, Walkersville, MD, USA and cultured in Fibroblast basal Medium-2 (Lonza, Walkersville, MD, USA) with supplements (FGM-2 bullet kit, Lonza, Walkersville, MD, USA). The NHLFs were passaged and used till passage 4. Human Peripheral blood mononuclear cells (PBMCs) were isolated from whole blood of healthy volunteers from New York Blood Center upon obtaining informed consent and after Institutional Review Board (IRB) approval. were isolated by Ficoll separation and were maintained in RPMI-1640 medium (Gibco, Grand Island, NY, USA) with 10% FBS (Gibco, Grand Island, NY, USA) and 1% penicillin/streptomycin (Gibco, Grand Island, NY, USA) prior to the experiment. All the cells were incubated in 5% CO_2_ supply at 37 °C.

### 2.2. Bacterial Strains

Cultures of recombinant *Bacillus Calmette–Guérin* expressing mCherry (BCG-mCherry), BCG, wild type *Mycobacterium tuberculosis* H37Rv (H37Rv), hypervirulent (HN878) and hyper-immunogenic (CDC1551) clinical isolates of Mtb were grown to log phase in Middlebrooks 7H9 broth with 0.05% tween 80 and 10% oleic acid–albumin–dextrose–catalase enrichment (OADC) at 37 °C under continuous shaking and frozen at −80 °C. Bacterial CFU/mL was determined by plating serial dilutions of multiple stocks on Middlebrook 7H10 agar plates and incubating at 37 °C. These stocks were later used for infection of TB spheroid at different multiplicities of infection (MOI).

### 2.3. Three-Dimensional TB Spheroid Formation

The wells of Aggrewell™ 400, 24-well spheroid-forming plates (Stemcell Tech, Vancouver, Canada) were used according to the manufacturer’s instructions. After surface treatment with anti-adherence rinse solution (Stemcell Tech, Vancouver, Canada), the THP-1 monocytes were seeded at 1 × 10^6^ cells/well. To produce TB spheroid, BCG-mCherry/Mtb H37Rv/CDC1551/HN878 were used at various MOIs (range 0.1–10). To produce THP-1 spheroids, the cells and bacteria were mixed together and then added to the Aggrewell™400 plate. The bacteria–cell suspension (total volume of 1000 µL/well) was then centrifuged at 1300 rpm for 5 min. For the formation of activated TB spheroids, cells in the microwell were cultured in the presence of 100 ng/mL Phorbol 12-myristate 13-acetate (PMA, Sigma Aldrich, St. Louis, MO, USA), for 24 h and then switched to standard RPMI media without PMA. A 70% media change (700 µL) was performed every day to maintain the TB spheroid cultures. In the case of non-activated spheroids, PMA treatment was not performed and, mentioned in the manuscript accordingly. Thus, for all the experiments involving THP-1 cells, we used PMA-treated spheroids unless specified otherwise. For harvesting the spheroids for downstream processing such as fluorescent staining, the media solution was then pipetted out in the spheroid wells gently to create a shear. The spheroids were displaced from the microwells and then they were pipetted out. The spheroids were allowed to settle down by the force of gravity for 15 min. The medium was gently removed, and the spheroids were washed twice with 1× DPBS. The spheroids were then used for staining. For the formation of PBMC spheroids, the same procedure outlined for THP-1 cells was used.

### 2.4. Three-Dimensional PBMC-Stroma Co-Cultures

PBMC co-culture spheroids were produced by mixing PBMCs and NHLFs at desired ratios and adding to the anti-adherence solution treated Aggrewell™ 400 plates. Briefly, 1 × 10^6^ PBMC cells and 0.1 × 10^6^ NHLFs and centrifuged at 1300 rpm for 5 min. This cell number is the default condition used in the experiment, unless otherwise indicated. For the experiment with the optimization of cell numbers to form co-culture spheroids (Figure S14), we used 1 × 10^6^ PBMC cells and 0.1 × 10^6^ NHLFs and 5 × 10^6^ PBMC cells and 0.5 × 10^6^ NHLFs. In the case of BCG mCherry infection, an MOI of 1 was used for creating the infection model. The MOI was calculated based on the number of PBMCs in the well. For formation of PBMC spheroids, we did not use PMA treatment. Similarly, to form the PBMC spheroids, we cultured the cells in RPMI media with 10%FBS but without Pen/Strep.

### 2.5. Staining and Fluorescent Microscopy

#### 2.5.1. Live Dead Staining

Zeiss Axiozoom microscope was used for imaging samples unless specified. The viability of THP-1 TB spheroids that were harvested on day 3 and day 5 and was assessed using Live-Dead cell viability assay as per the manufacturer’s recommended protocol (Invitrogen/ThermoFisher Scientific, Waltham, MA, USA). Briefly, the TB spheroid were washed with DPBS once and incubated for 30 min with Calcein AM (2 μm) and ethidium homodimer (4 μm)/propdium iodide (Nexcelom biosciences, Lawrence, MA, USA) and imaged. Whenever Calcein AM-PI staining was used, the spheroids were infected with BCG without mCherry.

For staining the cells in spheroids with DAPI, unfixed live spheroid cultures were used. Spheroids were stained with 1:1000 DAPI (Thermofisher Scientific, Waltham, MA, USA) for 30 min at room temperature. Following this, the spheroids were washed twice with PBS and then imaged. Since the live cultures were used, DAPI was used to assess cells with a permeable cell membrane/dead cells within the spheroids, as reported in a previously published article by Wallberg et al. [35].

#### 2.5.2. Hypoxia Staining

The hypoxia gradient across TB spheroid was detected using the Image IT hypoxia red stain according to the manufacturer’s protocol (ThermoFisher Scientific, Waltham, MA, USA). A 5 μm solution of Image-iT™ Red Hypoxia Reagent was added to the growth media and incubated for 60 min at 37 °C. The samples were washed 3 times with PBS to remove the residual stain. Following this, the TB spheroid were harvested and imaged. Single TB spheroid were analyzed using MATLAB (2019) to display surface plot intensities.

#### 2.5.3. Nile Red Staining

For Nile red staining, spheroids were harvested and washed with DPBS twice. Following this, they were fixed with 4% paraformaldehyde for 1 h at room temperature. Following this, they were washed and stained with 100 ng/mL Nile red stain (Sigma Aldrich, St. Louis, MO, USA) for 1 h at room temperature. The spheroids were washed with DPBS to remove additional stain and imaged.

### 2.6. Generation of Spheroid Sections and Staining

On day 5 post infection, TB spheroids were washed with PBS, harvested, and fixed using 4% paraformaldehyde for 45 min and carefully collected in 1.5 mL centrifuge tube and paraffin-embedded for sectioning. Then, 5 µm serial sections were mounted on glass slides, and de-paraffinized for H&E staining and AFB staining. Slides were analyzed by using the Nikon Microphot microscope with NIS Elements software (Nikon, Melville, NY, USA).

For immunofluorescent staining, the sections were incubated in 10 mM citrate buffer (pH 6) in a steam chamber for retrieving antigens. Immunostaining was performed by adding blocking solution (5 mg/mL BSA in PBS) for 30 min at room temperature, followed by primary antibodies IBA1 (Abcam, Cambridge, UK) and DyLight 650 conjugated HIF-1α (Invitrogen) with 0.5 μg/mL in blocking solution and incubating the sections overnight at 4 °C. After washing with PBS, sections were incubated at room temperature for 1 h in the dark with Alexa-488 labeled secondary antibody (0.5 μg/mL) (Abcam, Cambridge, UK) diluted in blocking solution. Slides were washed with PBS and mounted using Fluoroshield mounting medium containing DAPI (Abcam, Cambridge, UK). Images were acquired using an Axiovert 200 M inverted fluorescence microscope (Zeiss, Oberkochen, Germany) and a Prime sCMOS camera (Photometrics, Tucson, AZ, USA) controlled by Metamorph image acquisition software (Molecular Devices, San Jose, CA, USA). Image analysis was performed using software ImageJ/Fiji.

### 2.7. Multiplex Cytokine Analysis

Day 5 culture supernatants of TB spheroid were filtered through 0.2 μm filter membrane and stored at −80 °C until use. Human cytokine 27-plex assay kit was used as per manufacturers’ protocol (Bio-Rad, Hercules, CA, USA). Briefly, 1X magnetic beads were coated in the 96-well plate provided with the kit followed by the addition of blank standards and samples. Streptavidin-PE was added. After each step, wells were washed with buffer provided in the kit to ensure the removal of non-specific bindings. Samples were suspended in assay buffer after washing steps and read on Bio-plex 200 system (Bio-Rad, Hercules, CA, USA). Cytokine levels for infected TB spheroid were normalized with values from uninfected TB spheroid.

### 2.8. Mass Spectrometry Imaging

Spheroids were embedded in 10% gelatin, snap frozen and stored at −80 °C. Upon analysis, serial sections were taken at 10 µm using a Leica cryostat. Bright-field and immunofluorescent (IF) images of the spheroids were acquired using a Ti2-Epi Fluorescence Microscope (Nikon) prior to mass spectrometry imaging. Matrix deposition was carried out using the HTX M5 sprayer (HTX Technologies LLC, Chapel Hill, NC, USA). Then, 9-aminoacridine (9AA) at a concentration of 10 mg/mL was deposited onto the spheroid sections using flow rate of 160 µL/min and a temperature of 60 °C for a total of 14 passes using the crisscross setting. MS images were acquired using a Bruker SolariX 7T FT-ICR mass spectrometer (Bruker Daltonics, Billerica, MA, USA) equipped with a dual ESI/MALDI ion source and with a smartbeam II Nd:YAG laser, 355 nm. Data were acquired using the small laser setting and the oversampling method [36], which resulted in a pixel size/image resolution of ~20 µm. The instrument was operated in the negative ion mode over the mass range *m*/*z* 150–3000. Following data acquisition, the slides were stained with hematoxylin and eosin (H&E) and scanned using the Pannoramic DESK II DW scanner (3DHISTECH, Budapest, Hungary).

### 2.9. MALDI-MSI Data Analysis

Data were processed using the SCiLS Lab MVS, version 2020a Pro (Bruker Daltonics, Billerica, MA, USA). Control and infected spheroids were incorporated into a single file for lipidomic signal intensity comparison. The ion maps were processed using the Viridis color scale. For infected spheroids, the IF and H&E merged images were imported into the software for a direct overlay of lipid data with cell distribution and infection status. For the control spheroids, the H&E-stained section was imported and overlaid with the MSI data for correlation with cell regions.

### 2.10. Transmission Electron Microscopy

Spheroid samples were harvested on day 5 time point and suspended in buffered glutaraldehyde solution as outlined in the previously published paper by Rhode et al. [37]. The spheroids were further fixed in 1% osmium tetroxide solution. Following this step, the spheroids were dehydrated using ethanol and propylene series and treated with Spurr’s resin and the blocks were polymerized. Then, 70 nm sample sections were generated and were imaged using FEI Tecnai 12 Bio-twin transmission electron microscope.

### 2.11. Drug Treatment

The TB spheroids infected with BCG mCherry were cultured for 3 days in Aggrewell plates and then used for drug treatment studies. Isoniazid (INH), Rifampicin (RIF) and DMSO were purchased from Sigma Aldrich, St. Louis, MO, USA. A combination of 0.625 µM INH and 0.195 µM RIF/DMSO control was used.

### 2.12. Bacterial Colony Formation (CFU Assay)

To quantify mycobacterial survival in TB spheroid after culture and/or drug treatment, the CFU assay was performed. TB spheroids were suspended in 0.05% Triton X-100 lysing solution and incubated at 37 °C for 5 min. Suspension was collected in a centrifuge tube and vortexed 5 times for 10 s with intermittent 30 sec incubation on ice. Suspension was then centrifuged at 10,000× *g* for 5 min at room temperature, and the pellet was suspended in 100 μL of 7H9 broth. Serial dilutions were made in Middlebrook 7H9 media containing 0.05% tween-80, and 10 μL samples were spotted in triplicates on the Middlebrook 7H10 agar plates. Colonies were counted after 14 days of incubation at 37 °C and CFU/mL was determined.

## 3. Results

### 3.1. Modelling the TB Spheroid Infection In Vitro

**Figure 1:**
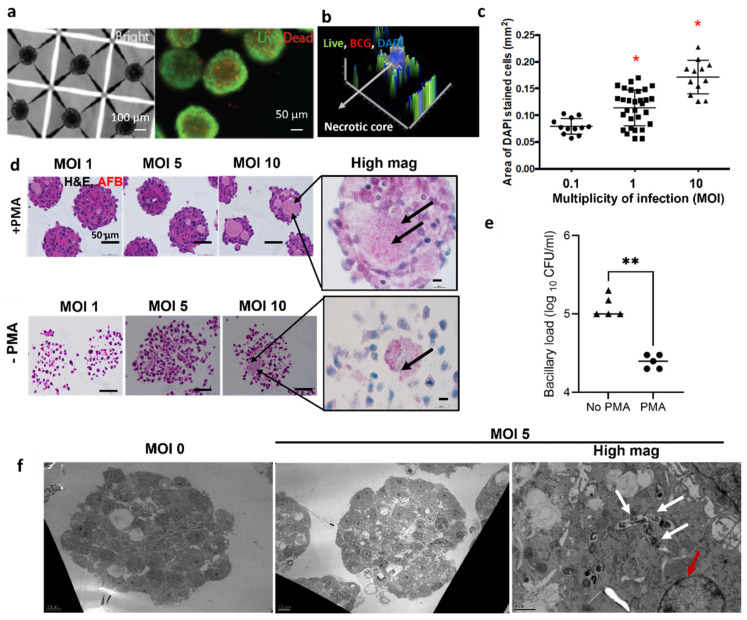
Modelling the TB spheroid infection in vitro. (**a**) Imaging of THP-1 TB spheroid using bright field, live (Calcein AM, Green) and dead (Ethidium homodimer, red) stain. (**b**) A 2.5D intensity projection J774 spheroid infected with BCG mCherry, Calcein AM and DAPI (blue). Arrows indicate presence of DAPI stained cells in the center of TB spheroid (**c**) area of DAPI + dead cells in TB spheroids formed with J774 infected with increasing MOI of BCG mCherry quantified via Image J analysis. * Represents *p* < 0.05, by one-way ANOVA. (**d**) Images of 5 µm sections of TB spheroid cultured with and without phorbol 12-myristate 13-acetate (PMA), stained with H&E (host cells), and acid-fast bacilli (AFB, bacteria). Arrows in the high magnification images show the AFB (Red) staining for bacilli. (**e**) Bacterial CFU/mL estimated by culturing TB spheroid lysates (*n* = 5 wells/condition). ** represents *p* < 0.01 by Student *t*-test. (**f**) Transmission electron microscope images of spheroid sections with and without BCG mCherry at MOI 5. White arrows in the high magnification image show the localization of BCG within a macrophage. Red arrow shows the presence of necrosis-like cells in the spheroid in the high mag image.

Robust TB spheroids were formed successfully, using different initial cell seeding densities, for human and mouse monocyte/macrophages ranging from 1–2.4 × 10^6^ cells/well (THP-1, J774 cells) and human T cells 1.2–6 × 10^6^ cells/well (Jurkat) (Figures S1b,c and S2a,b). While the J774 and Jurkat T cells were used for initial validation experiments, we chose THP-1 cells because they formed tight spheroids and can be used in conjunction with PMA activation. We observed an increase in spheroid size with the increase in number of cells seeded/well (Figures S1c and S2a). In the case of THP-1, we also observe heterogeneity in spheroid size distribution but a similar average spheroid diameter of 150–200 µm as shown in Figure S1c owing to the tight range of cell seeding numbers chosen for optimization. Thus, for further experiments, we chose 1 × 10^6^ cells/well as an optimal cell seeding condition. Spatial analysis of cell viability of uninfected THP-1 spheroids demonstrated the presence of live cells in the periphery and some dead cells in the center (Figure 1a), presumably due to diffusion limitations in 3D models of ~200 µm in diameter. By co-incubating THP-1 with *M. bovis* BCG, we created TB spheroid with a centralized core of dead cells with BCG (Figure 1b). Based on previously published studies, DAPI has been used to stain for dead cells when used on un-fixed samples [35,38]. In order to address potential issues with non-specific staining of DAPI and its accumulation, we used Calcein AM staining as an orthogonal stain to check cell viability in the same samples. We observed that an increased ratio of bacteria to host cells from multiplicity of infection (MOI) from 0.1–10, led to a significant increase in the DAPI stained cells, suggesting an increased host cell death (Figure 1c). To evaluate the time-dependent difference in host-cell death, activated spheroids were harvested on day 3 and day 5 and stained with Calcein AM and PI (Figure S3b) and H&E (Figure S3c). Spheroids harvested on day 5 showed increased host-cell death marked by increase in propidium iodide cell-stained cells (Figure S3b) when compared to day 3 spheroids and demonstrating time-dependent differences in interaction between host and pathogen.

Activation and differentiation of THP-1 monocytes into macrophages with phorbol 12-myristate 13-acetate (PMA) was critical for containing bacterial growth. Histological evaluation of PMA-treated TB spheroid infected with BCG mCherry displayed a compact spheroid with acid-fast bacilli positive bacteria localized within dead cells (Figure 1d). In the non-activated group, cells were loosely packed (Figure 1d). Similarly, in H37Rv infected TB spheroids, the activated group produced tightly packed spheroids and the non-activated group produced loose spheroids which disintegrated during sectioning (Figure S4). To determine the effects of activation on bacterial growth, a colony forming unit assay (CFU) was performed. A 10-fold reduction in bacterial proliferation was observed in the activated group, when compared to the non-activated group (Figure 1e). The data suggest that spheroid structure is tightly controlled by monocyte activation and differentiation into macrophages.

To confirm the sub-cellular spatial localization of the bacteria within the spheroids, we also performed transmission electron microscopy on spheroid sections as shown in Figure 1f. Ultrastructure observations indicate that there is higher cell death in the TB spheroids when compared to control non-infected group. Based on the ultrastructure observation, we identified the presence of necrotic cells within the TB spheroids as indicated in Figure 1f and Figure S5. In addition, we observed that multiple bacteria reside within individual vacuoles (Figure 1f).

### 3.2. TB Spheroid Are Hypoxic and Promote Proinflammatory Milieu

**Figure 2:**
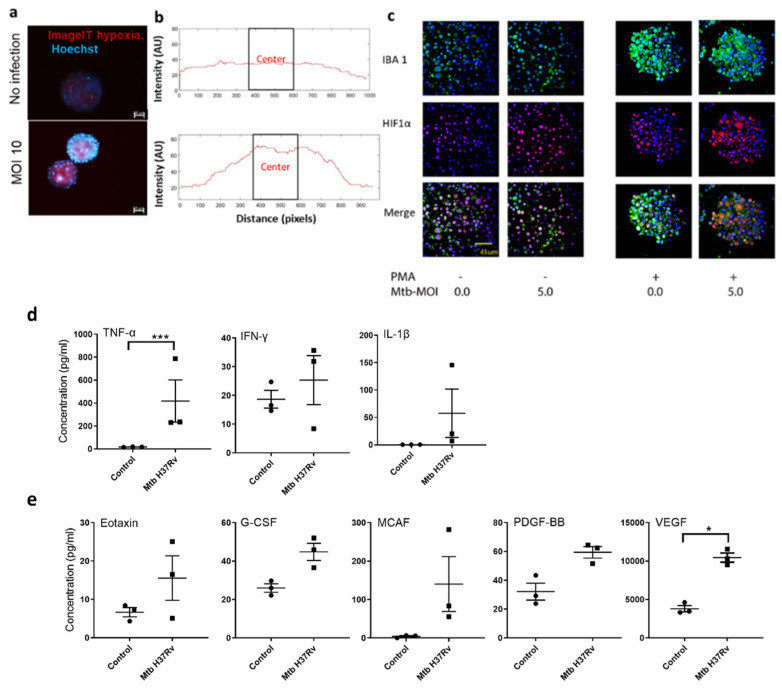
TB spheroids are hypoxic and pro-inflammatory (**a**) Fluorescent images of activated TB spheroid infected with BCG and cultured for 3 days. TB spheroids were stained with hypoxia probe (red) and Hoechst (blue). (**b**) The panels on the right show representative image of hypoxic probe intensity along the diameter of TB spheroid (size: 150–200 µm), determined using MATLAB (Code is provided in Appendix A). For (**a**,**b**), images on top represent uninfected control groups, and the bottom represents TB spheroid infected with MOI 10 (BCG to macrophages). (**c**) Immunofluorescence-stained sections of activated (PMA) and non-activated TB spheroid infected with MtbH37Rv at MOI-5 and probed with antibodies specific to macrophages (IBA-1, green; top panel) or hypoxia-inducible factor (HIF-1a red; middle panel) imaged at a timepoint of 5 days post harvesting. The bottom panel shows the merged image. (**d**,**e**) Cytokine and growth factor profiles of Mtb H37Rv infected TB spheroid after 5 days in culture measured using multiplex cytokine analysis. Each dot in d and e represents the mean (average) value of an experiment with *n* = 3 wells per cytokine/growth factor estimation. Data presented are mean ± SEM of three experiments. (* *p* < 0.05; *** *p* < 0.005 when compared to non-infected controls, paired Student *t*-test).

In vivo, granulomas are densely packed with host cells and are hypoxic at the center [39,40]. Mtb-infected macrophages express HIF1α, under normoxic conditions [41]. A hypoxia probe showed an increased stain intensity in BCG-infected TB spheroids when compared to the non-infected controls on spheroids harvested on day 3 time point (Figure 2a). Surface intensity plot across the diameter of a single TB spheroid demonstrated a gradient in hypoxia from the periphery (low intensity) to the center (high intensity) in the BCG-infected group. (Figure 2b). The link between monocyte activation, differentiation, infection, and hypoxia, was also validated using pathogenic Mtb H37Rv. After 5 days of co-culture, TB spheroids were harvested and stained for hypoxia (HIF1α) and macrophage activation (IBA-1), as shown in Figure 2c. As expected, the spheroids of PMA-activated macrophages had a significantly higher percentage of cells expressing IBA1, HIF1α and colocalization of these two molecules. Although these activated spheroids demonstrated an increase in HIF1α upon Mtb infection, the difference was not significant. Similarly, the percentage of cells expressing IBA1 and those colocalizing IBA1 and HIF1α were comparable between Mtb-infected and non-infected activated spheroids (Figure S6). Interestingly, in the non-activated spheroids, Mtb-infection increased the percentage of IBA1, HIF1α-expressing and colocalizing cells, with significantly higher HIF1α-expressing cells, suggesting that Mtb-infected macrophages induce hypoxia (Figure S6).

Next, we performed multiplex cytokine analyses on supernatants from an Mtb H37Rv-infected group and no infection control, as shown in Figure 2d,e. With Mtb H37Rv infection, we observed an increase in TNF-α when compared to non-infected control. IFN-γ, IL-1β were induced at higher levels in both groups. Hypoxia-mediated growth factors, VEGF and GCSF were also upregulated in Mtb H37Rv infected TB spheroids (Figure 2e). Thus, our TB spheroid produced proinflammatory cytokines in response to mycobacterial infection.

### 3.3. TB Spheroids Display Extensive Lipid Deposition

**Figure 3:**
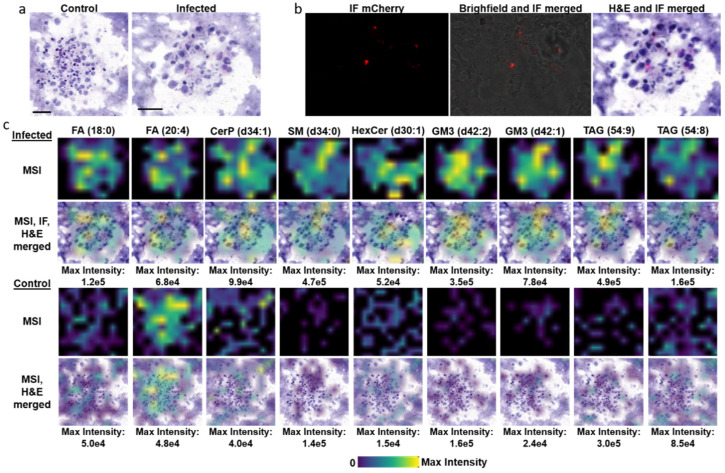
Mass spectrometry imaging (MSI) of TB spheroids detects lipidomic alterations that correlate with human TB and animal models of infection. (**a**) Hematoxylin and Eosin (H&E) stained spheroids treated with PMA used for MSI. (**b**) Immunofluorescence (IF) of the mCherry BCG infected spheroid (left panel), brightfield and IF merged image (middle panel), IF of mCherry BCG and the corresponding H&E of the same spheroid. (**c**) MSI data of lipids detected in infected vs. control spheroids (top panels). MSI lipid data overlaid with the IF-H&E merged images correlating lipid data with cell infection status (bottom panel of infected spheroids). MSI lipid data detected in control spheroids overlaid its corresponding H&E section (bottom panel). FA, fatty acid; CerP, ceramide-1-phosphate, SM, sphingomyelin; HexCer, hexosylceramides; GM3, monosialodihexosylganglioside; TAG, triacylglyceride. The scale bars are each 100 um.

Human TB granulomas are characterized by lipid deposition and accumulation that lead to foam cell formation in macrophages and dendritic cells [42,43]. Triacylglycerol, cholesterol, fatty acids, and sphingolipids have all been shown to accumulate within these cell types following infection [42,43,44]. We validated the presence of lipids using Nile red staining in BCG infected spheroids as shown in Figure S7. We also demonstrate an increase in lipid deposition in BCG-infected TB spheroids compared to non-infected controls (*n* = 4 replicates) using mass spectrometry imaging (Figure 3 and Figure S8–S12). Representative images demonstrate dysregulated lipid metabolism that correlates with those observed in human granulomas. Increases in fatty acids, sphingolipids and triacylglycerols were all detected in infected TB spheroids when compared to controls (Figure 3c). The changes in triacylglycerol signal between the non-infected and infected spheroids were 1.6-fold for TAG (54:9) and 1.9-fold for TAG (54:8). With the exception of FA (20:4), which demonstrated only a 1.4 fold increase, the remaining lipids presented in Figure 3 including the sphingolipids and FA (18:0) experienced 2.5- to 3.5-fold increases in the BCG-infected spheroids. Interestingly, by overlaying the MSI data with its corresponding H&E-stained section, we show that neighboring cells within the spheroids demonstrate differences in lipid accumulation, which is enabled by the at/near cellular resolution of the method used as most macrophages within the spheroid are ~20 µm in diameter. This is evident by the signal intensity differences that are mapped to individual cells within the spheroids (Figure 3c top panel and Figures S8–S12 right panels). There was variability in the detection of arachidonic acid in the four replicates of the control and infected spheroids and this is potentially attributed to using PMA for activation. Finally, by overlaying the TB spheroid IF mCherry image with its H&E and MSI data, we can directly map lipid accumulation to cell infection status. This has never been achieved before and affords the opportunity to directly investigate dysregulated host lipid metabolism in relation to the infection status of the immune cell.

### 3.4. TB Spheroids Display Bacterial Strain Dependent Response to Anti-TB Drugs

**Figure 4:**
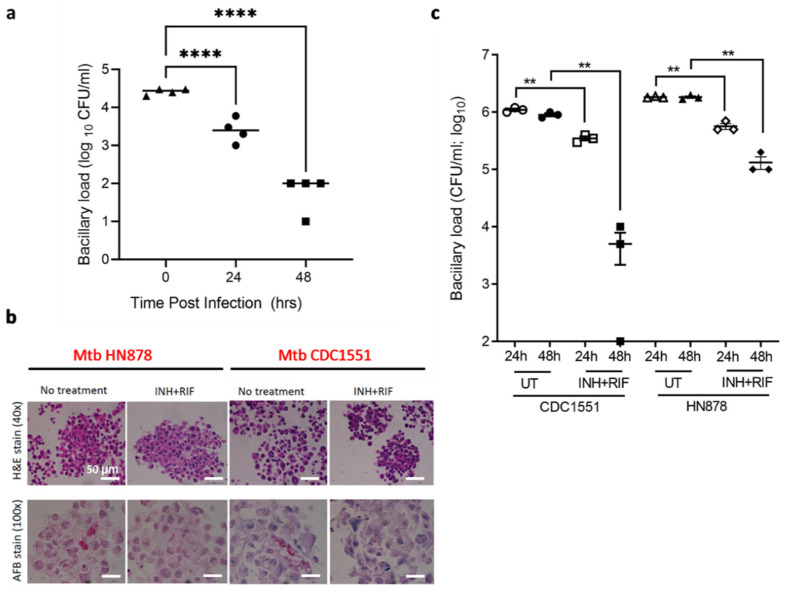
TB spheroids show mycobacterial virulence dependent response to anti-TB drugs (**a**) Estimation of bacterial colony-forming units (CFU/mL) from lysates isolated from PMA-treated TB spheroids infected with BCG mCherry post-treatment with INH and RIF combination at 0, 24 and 48 h time points. (**b**) Images of TB spheroid sections with HN878 and CDC1551 infection, stained with H&E (host cells) and AFB (bacteria). (**c**) CFU/mL determined from TB spheroid infected with clinical isolates Mtb HN878 and CDC1551 and treated with DMSO (UT) and INH+RIF combination. ** *p* < 0.01; **** *p* < 0.0001 by one-way ANOVA followed by Tukey’s multiple comparisons test for comparisons of clinical isolates from treated group compared with the respective untreated group. Data and mean from *n*  =  3 wells, comprising of approximately 1200 TB spheroid/well.

To determine whether the TB spheroid model can be useful for drug screening, we evaluated the response to first-line anti-TB drugs, isoniazid (INH) and rifampicin (RIF) [45,46,47] in activated TB spheroids infected with BCG mCherry. The CFU assay performed on lysates obtained from 0, 24, and 48 h INH and RIF combination treatment (0.625 µm INH and 0.195 µm RIF) show that bacterial CFU decreases with the time of exposure to the drugs (Figure 4b).

Finally, to study the role of bacterial virulence on drug response, we used two different Mtb strains, HN878 and CDC1551 with different virulence [48,49,50]. The CD1551 and HN878-infected TB spheroid were treated with a combination of INH and RIF or DMSO (control). With INH-RIF treatment, CDC1551 shows a dramatic decrease in AFB staining (Figure 4b). Bacterial CFU assay was performed from lysates obtained at 24 and 48 h, as shown in Figure 4c. Untreated TB spheroids of HN878 infection demonstrated a higher CFU/mL (~1.8 × 10^6^) when compared to CDC1551 (~1.1 × 10^6^) and correlates with the results from AFB staining. These results show that the TB spheroid formed with two different Mtb strains has differential response to anti-TB drugs.

### 3.5. Formation of PBMC-NHLF Co-Culture TB Spheroids

**Figure 5:**
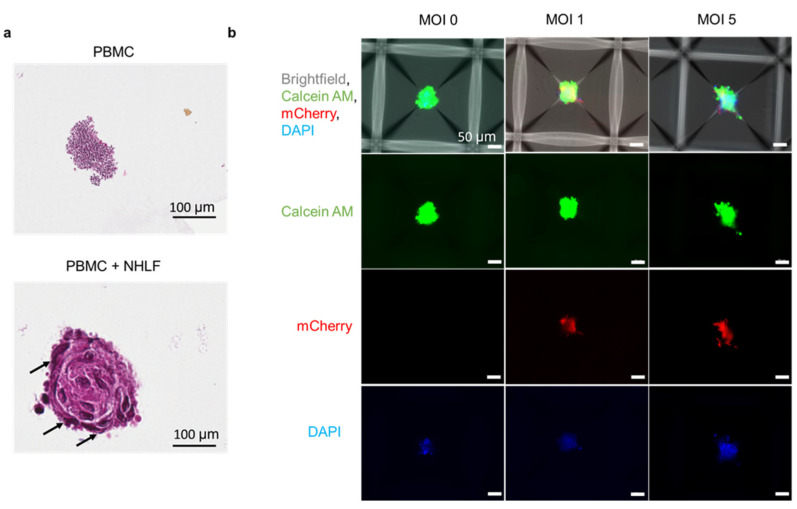
Formation of 3D co-culture spheroids (**a**) H&E-stained PBMC monoculture and NHLF co-cultured spheroids harvested on day 5 in culture. Arrows indicate fibroblast cells present in the periphery based on morphology. (**b**) Imaging live PBMC co-cultured spheroids with varying MOI of BCG mCherry using bright field, live (Calcein AM, Green) and dead (DAPI, blue) stain (*n* = 2 wells/condition, ~1200 spheroids/well).

In vivo, tuberculosis granulomas are characterized by the presence of immune cells such as macrophages, γδ (gamma)(delta) T cells, NK cells, neutrophils, and eosinophils. Therefore, we adapted the spheroid model to peripheral blood mononuclear cells to model the heterogeneous cell population to some extent. PBMC spheroids were infected with BCG mCherry at MOI 1 as shown in Figure S13a. However, these spheroids were loosely packed and disintegrated during sectioning and H&E staining (Figure S13b). To evaluate whether the addition of fibroblast-like cells stabilizes the granulomas, we co-cultured PBMCs and normal lung fibroblast (NHLFs)/mesenchymal stem cells (MSCs) as shown in Figure S15. The addition of NHLFs/MSCs enables the formation of tight spheroids as shown in Figure 5a. In addition to this, we observed that the fibroblasts localized in the periphery of the spheroids and mimicked the fibrotic cuff of mature TB granulomas (Figure 5a). Next, we evaluated the effect of increasing MOIs of BCG mCherry on cell death within the heterogeneous PBMC-NHLF co-culture spheroids (Figure 5b). We observed the presence of dead cells in the center, marked by DAPI positive cells. These results collectively indicate that heterotypic spheroids can be established using the co-culture method and the addition of fibroblast-like cells aid in stabilizing immune granulomas and enable the formation of tight spheroids.

## 4. Discussion

Granuloma formation is one of the hallmarks of TB, and it is characterized by a central core of necrotic, Mtb-infected macrophages, surrounded by other immune cell types that prevent the mycobacterial spread. These granulomatous structures remain an enigma despite decades of research to understand the containment of Mtb infection and reactivation of disease. To decode this, we generated 3D spheroids to capture the aspect of self-assembly of cells into a single cluster and providing a three-dimensional structure. We demonstrate the presence of necrotic nuclei, which are amorphous and lack clear nuclear condensation, which is much more marked in programmed cell death. From the gross pathological observation from TEM, we infer that the majority of the cell death is necrotic (Figure 1f and Figure S5). We demonstrate that mycobacteria reside within the dead macrophages via cell staining and histological assessment, providing underlying evidence for the presence of caseum (Figure 1b–d) and necrosis-like morphology (Figure 1e and Figure S5). This primary validation can be further strengthened in future by harvesting cells from spheroids and characterizing the type of cell death within the TB spheroids using quantitative methods such as flow cytometry. Based on the pathological observation, we identified that bacteria reside within the macrophage cells, and we did not observe any extracellular bacilli (Figure 1f and Figure S5).

Using the TB spheroids, we identified an important aspect of structural differences of spheroids produced using activated vs, non-activated monocytes. H&E and AFB staining demonstrated that monocyte activation and differentiation is critical for the formation of tightly controlled TB spheroid structures (Figure 1d) and restricting bacterial growth as seen in the CFU assay (Figure 1e). A similar result was observed by Leemans et al., where depletion of activated macrophages in mouse TB models led to bacillary growth [51]. It is critical to understand that the CFU assay values reflect both intracellular bacteria and extracellular replication, after harvesting from spheroids. However, the control (no-PMA) and PMA-treated samples were cultured at the same time, using the same conditions. It should be noted that, relative to the control group, the PMA-treated group is hypothesized to have a lower starting bacterial concentration. As a result of this, the value of CFU for the PMA-treated group is lower at the end of this assay. In addition to the control of spheroid structure, activation of monocytes and differentiation into macrophages is responsible for inducing hypoxia in TB spheroid, which is demonstrated via hypoxia probe (Figure 2a,b) and HIF1α (Figure 2c) staining. These data suggest that mycobacterial infection induces hypoxia in TB spheroid beyond that of oxygen transport limits in 3D spheroids. While we observed the hypoxic probe intensity gradient in Figure 2a,b, we did not observe the spatial variation in HIF1α staining in Figure 2c. This could be because of the differences in the time points used for analysis of these two studies and the differences between using BCG vs. Mtb H37Rv and the use of two different methods to characterize hypoxia. From the literature available on characterizing spheroids, some of the common methods for staining for hypoxia are using ImageIT probe [52], pimonidazole [53], Ru-dpp [54] and HIF1α [55]. The first three methods (ImageIT probe staining, pimonidazole and RU-DPP) are used on live spheroids and they provide information about the oxygen levels on spheroids before any downstream processing such as fixing/sectioning. In terms of directly comparing HIF1α staining and ImageIT probe staining, a few studies in the literature in the field of oncology have compared the spatial localization of the ImageIT hypoxia probe and HIF1α staining, and the results are variable. For instance, Gilmore et al. validated different methods to characterize hypoxia and found that HIF1α and Image IT probe spatial localizations correlate with each other [56]. On contrary, a study using patient tissue sections demonstrated that pimonidazole localization was confined to the central necrotic region in the center of the tissue, while HIF1α localization was not confined to the center [57]. It was found that there are key factors that influence co-localization of HIF1α such as time of sample analysis. For instance, Sobanifar et al. demonstrated using frozen cervical carcinoma xenograft sections that were harvested after 90 min post pimonidazole injection vs. 48 h post pimonidazole treatment and found that, at the 90 min time point, there was 80% co-localization of HIF1α and pimonidazole vs. a 32% co-localization at the 48 h time point [58]. This shows that time of analysis impacts the correlation between the two markers. It is critical to note that the ImageIT hypoxia stain shown in Figure 2a is imaged on 3-day time point, whereas the HIF-1α staining and IBA localization shown in Figure 2c is a 5-day time point.

Hypoxia is also known to mediate proinflammatory cytokine secretion and immune cell recruitment via IL-1β and TNF-α. Activated M1 macrophages produce proinflammatory cytokines that confer host protection [59,60]. TB patients have elevated plasma levels of proinflammatory TNF-α, IL-1α, IL-1β, IFN-γ and IL-6 [61,62]. We observed an increase in TNF-α when compared to the non-infected control in the TB spheroid (Figure 2d). However, we did not find any statistically significant difference in other proinflammatory cytokines such as IL-1β and INF-γ when compared to the non-infected group. This can be attributed to the inherent increase in these cytokines with the PMA activation. These results demonstrate that the TB spheroid model mirrors hypoxia, and secreted factors found in vivo in animal models and humans.

Dysregulated lipid metabolism is a hallmark of human TB granuloma development that has also been recapitulated in animal models [42,43,44,63,64]. This is a multifaceted phenomenon, infection of macrophages and dendritic cells drives the foam cell phenotype in these cells and in bystander uninfected cells, which is a characteristic feature of necrotizing and caseating lesions. Triacyl glyceride and cholesterol accumulation within these cells had been well documented [65]. Glycosphingolipid accumulation and dysregulation of genes involved in sphingolipid and glycosphingolipid production and metabolism have also been reported in human TB granulomas and in vitro infection studies [43]. Additionally, the accumulation of cholesterol and sphingolipids in macrophages, coupled with the ability of Mtb to prevent lysosomal maturation, is a phenotype that mimics the profile of lysosomal storage diseases such as Gaucher and Niemann-Pick diseases [66,67]. A recent in vitro study supported this notion by demonstrating infection of macrophages with Mtb or supplementing the media with mycolic acids produced a Niemann-Pick C phenotype [68].

Mass spectrometry imaging data from our TB spheroid model demonstrates a dysregulated lipid phenotype that correlates with those observed in animal and human TB granulomas (Figure 3). Immunometabolism has become a central focus of the investigation of TB pathology and treatment due to the realization that cellular metabolism is intricately linked with immune cell effector function and that these pathways can be manipulated for a more favorable outcome [69,70]. Studies have also demonstrated that macrophage phenotype is a product of both the immediate cytokine milieu, infection status and the ontogenic origin of the macrophage lineage. The Russell group has published extensively on dysregulated lipid and metabolic profiles of macrophages following infection, and recently demonstrated host permissiveness to infection was directly linked to macrophage origin and its pre-programmed metabolic status [71,72,73]. It should be noted that the description of macrophage polarization as M1 and M2 phenotypes does not reflect the spectrum observed in vivo and recently at least eight different activation states were detected [74]. While the characteristic structure of the granuloma is known, in which a necrotic core is surrounded by foamy macrophages, dendritic cells and a lymphocyte cuff, there is limited information on the spatial organization of the lipidomic and metabolomic phenotypes of these cells. To date, most studies have been carried out on single cells following in vitro infection or isolation of single cells following in vivo infection [75,76,77,78,79]. Our results demonstrate that the 3D TB spheroid model recapitulates the altered host lipid metabolism observed in macrophages from these studies and also provides a platform to study the metabolic immune response to infection (Figure 3c).

Finally, we investigated whether TB spheroids can be used to study drug response based on bacterial virulence using two different Mtb strains, HN878 and CDC1551 [48,49,50]. Mtb HN878 (a W-Beijing strain) accounts for 50% of infections in East Asia, and 13% worldwide [80,81]. Animal models using HN878 infection show exacerbated immunopathology, hypervirulence and high mortality rates. In contrast, Mtb CDC1551 shows reduced disease pathology and increased survival in mice, when compared to HN878 [82,83]. As expected, we observed that CDC1551-infected TB spheroids respond better than their HN878 counterparts, marked by the decrease in CFU (Figure 4c). The model can aid in reducing attrition of new therapeutics and improving current regimens by providing a more relevant in vitro model that will bridge the gap with in vivo studies, etc.

In vivo, TB granulomas are comprised of immune cells and fibroblasts. Recent studies have identified that there are two distinct types of fibrosis associated with TB, namely peripheral and centrally fibrotic granulomas. The peripheral granulomas form the characteristic fibrotic cuff and aid in controlling mycobacterial spread. The centrally fibrotic granulomas are characterized by extensive collagen deposition and are associated with a healing response, often containing no bacilli [84,85]. The difficulty to study fibrosis within in vivo models and the lack of in vitro models to depict this phenomenon makes it difficult to understand why fibroblasts are critical for formation of a compact granuloma structure. The ability of our system to culture multiple cell types together, as shown in Figure 5a demonstrates that this model can be used to study fibroblast recruitment and their impact on drug response in future. In addition, the spatial localization of fibroblast in the co-culture spheroid model is an interesting phenomenon that has never been studied. Thus, the heterogeneous TB spheroid model can facilitate future studies to identify the specific role of fibroblasts in controlling granulomas and their impact on drug response. Pioneering studies from orthogonal fields such as viral infections such as *Haemophiles influenzae* have investigated the micro-structure of the mucous layer and stromal compartments, shedding light into the microenvironment that facilitates colonization of bacteria [86]. The spheroid in vitro model provides us with the potential to not only generate co-cultures with several cell types but also generate potential viral-bacterial co-infections in future.

It is critical to state that the TB spheroid model is not designed to replace in vivo granulomas but to serve as a platform for initial validation of phenotypic aspects and drug response. Furthermore, the model can be developed to incorporate the cells from granuloma microenvironment to create granuloma mimetic 3D cultures in vitro. For high throughput applications, the assay can be scaled to 96- and 384-well formats. In the future, 3D TB spheroids can serve as potential intermediate models between lead identification from drug susceptibility testing to administration in animal models.

In summary, we established a novel 3D host–pathogen interactive platform that supports in vitro tracking of mycobacteria and host immune response. These miniaturized TB spheroid structures can be adapted to incorporate multiple immune cell types and screen anti-TB compounds in the future. This type of microwell arrays can be used in future to study impact of one spheroid over the neighboring spheroids. We do think that there will certainly be interactions in terms of secreted factors and metabolites. This type of study have been carried out using spheroids cultured within microwell arrays in the field of oncology [87,88] and liver modelling [89] by combining microfluidic technologies within spheroid platforms. We think that using confocal microscopy imaging and staining of spheroids, we can profile different cell types within the spheroids. This type of research on staining and profiling single cells within spheroids and organoids have been reported in the cancer field. In addition to this, with techniques such as MALDI MSI, which we have reported in the paper, spatial distribution of metabolites can be profiled within spheroid sections.

## Supplementary Materials

The following are available online at https://www.mdpi.com/article/10.3390/biomedicines9091209/s1, Figure S1: Formation of 3D spheroids using THP1 cells (a) Schematic for formation of TB spheroid. (b) Generation of THP-1 spheroids using different cell numbers. (c) Diameter of the spheroids estimated via Image analysis (n = 24–48 spheroids/condition). * denotes statistical significance (*p* < 0.05) when compared to 1 × 106 group using one-way ANOVA and Tukey post hoc test. Figure S2. Validation of the model to form spheroids using other immune cells. (a) Mouse macrophage and (b) Jurkat T. Diameter of n = 24–48 spheroids for each cell type are represented in the bar graphs. * denotes statistical significance (*p* < 0.05) when compared to 1 × 106 group using one-way ANOVA and Tukey post hoc test. Figure S3. Time dependent tracking of spheroids. (a) Brightfield images of THP-1 spheroids on day 3 and day 5; (b) Calcein AM (green-Live) and PI (red-Dead) stained spheroids on day 3 and day 5. (c) H&E-stained sections of BCG-infected spheroids harvested on day 3 and 5. Figure S4. Histological analysis of Mtb-infected spheroids. Images of 5 µm sections of Mtb infected spheroid cultured with and without phorbol 12-myristate 13-acetate (PMA), stained with H&E (host cells), and acid-fast bacilli (AFB, bacteria). Figure S5. TEM image of TB spheroid infected with BCG mCherry. Red arrows indicate necrotic cells. Figure S6. Quantification of co-localized IBA-1 and HIF-1α in non-activated and PMA-activated TB spheroid with or without Mtb infection. Number of cells positive for IBA-1 (green), HIF-1a (red) or both (orange red) were manually counted from images at 63× magnification. At least 50 non-necrotic, intact cells per field were counted from each spheroid and 3–4 spheroids from each of 3 sections were counted to obtain the data. The data were analyzed by one-way ANOVA with Tukey’s post analysis correction for multiple group comparison. * *p* < 0.05; ** *p* < 0.001; *** *p* < 0.0005. Figure S7. Nile red staining of THP-1 spheroids. Representative images of THP-1 spheroids infected with BCG mCherry (MOI 5) and no infection controls (MOI 0) stained with Nile red. Figure S8. MALDI MSI H&E staining in replicates. (a) Hematoxylin and Eosin (H&E) stained spheroid replicates used for MSI. (b) Immunofluorescence (IF) of the mCherry BCG infected spheroid replicates (top panel). Brightfield and IF merged images of the mCherry BCG infected spheroid replicates (middle panel). H&E and IF merged images used for MSI analysis (bottom panel). Scale bars are 100 µm. Figure S9. MSI data of fatty acids detected in control vs. infected spheroids. The overlaid MSI lipid data for the infected spheroids are with the mCherry IF-H&E merged images. Scale bars are 100 µm. Figure S10. MSI data of sphingolipids detected in control vs. infected spheroids. The overlaid MSI lipid data for the infected spheroids are with the mCherry IF-H&E merged images. Scale bars are 100 µm. Figure S11. MSI data of gangliosides detected in control vs. infected spheroids. The overlaid MSI lipid data for the infected spheroids are with the mCherry IF-H&E merged images. Scale bars are 100 µm. Figure S12. MSI data of triacylglycerides detected in control vs. infected spheroids. The overlaid MSI lipid data for the infected spheroids are with the mCherry IF-H&E merged images. Scale bars are 100 µm. Figure S13. PBMC aggregate formation. (a) Infection of PBMCs derived from two healthy donors with BCG mCherry at MOI 1. (b) H&E staining of harvested aggregate section. Figure S14. Formation of PBMC spheroids co-cultured with stromal cells. (a) Images of PBMCs co-cultured with NHLF and MSCs. (b) Quantification of average area of harvested spheroids. n = 2 wells/condition for all the groups except PBMC+ NHLF+ BCG mCherry (5× higher seeding), where n = 4 wells/condition were used and the average for each group was calculated based on ~1200 spheroids/well. Scale bar denotes 100 µm.

## Appendix A

**MATLAB Code for surface intensity plot (used in Figure 2** **b)**
Image = imread(‘Image.tiff’);
x = [0 size(Image,2)];
y = [size(Image,1)/2 size(Image,1)/2];
s = improfile(I,x,y);
figure
subplot (2,1,1)
imshow (Image)
hold on
plot(x,y,‘r’)
subplot (2,1,2)
plot(s(:,1,1),‘r’)
axis ([0 1000 0 80])
