# Supplementary material for: In Vitro Miniaturized Tuberculosis Spheroid Model
Source: Biomedicines. 2021 Sep 13;9(9):1209. doi: 10.3390/biomedicines9091209 (PMC8470281; doi:10.3390/biomedicines9091209)
Supplement: Supplementary file 1 [file biomedicines-09-01209-s001.zip › biomedicines-1317085-supplementary.pdf]

In Vitro Miniaturized Tuberculosis Spheroid Model

[1] Department of Biomedical Engineering, Rutgers, The State University of New Jersey, Jersey City NJ 08854, USA; fs386@scarletmail.rutgers.edu (S.M.); ars340@scarletmail.rutgers.edu (A.S.)
[2] Public Health Research Institute, New Jersey Medical School, Rutgers, The State University of New Jersey, Jersey City, NJ 07103, USA; poojasingh@uabmc.edu (P.S.); rk879@njms.rutgers.edu (R.K.); subbiase@njms.rutgers.edu (S.S.)
[3] Department Center for Discovery and Innovation, Hackensack Meridian Health, New Jersey, NJ 07110, USA; kelly.oneill@hmh-cdi.org (K.C.O.); claire.carter@hmh-cdi.org (C.L.C.)
[4] Department of Microbiology and Immunology, School of Veterinary medicine, Cornell University, Ithaca, NY 14853, USA; dgr8@cornell.edu
[5] Department of Medicine, Rutgers Biomedical Health Sciences, Rutgers, The State University of New Jersey, Jersey City, NJ 08854, USA
* Correspondence: biju.parekkadan@rutgers.edu

Supplementary figures

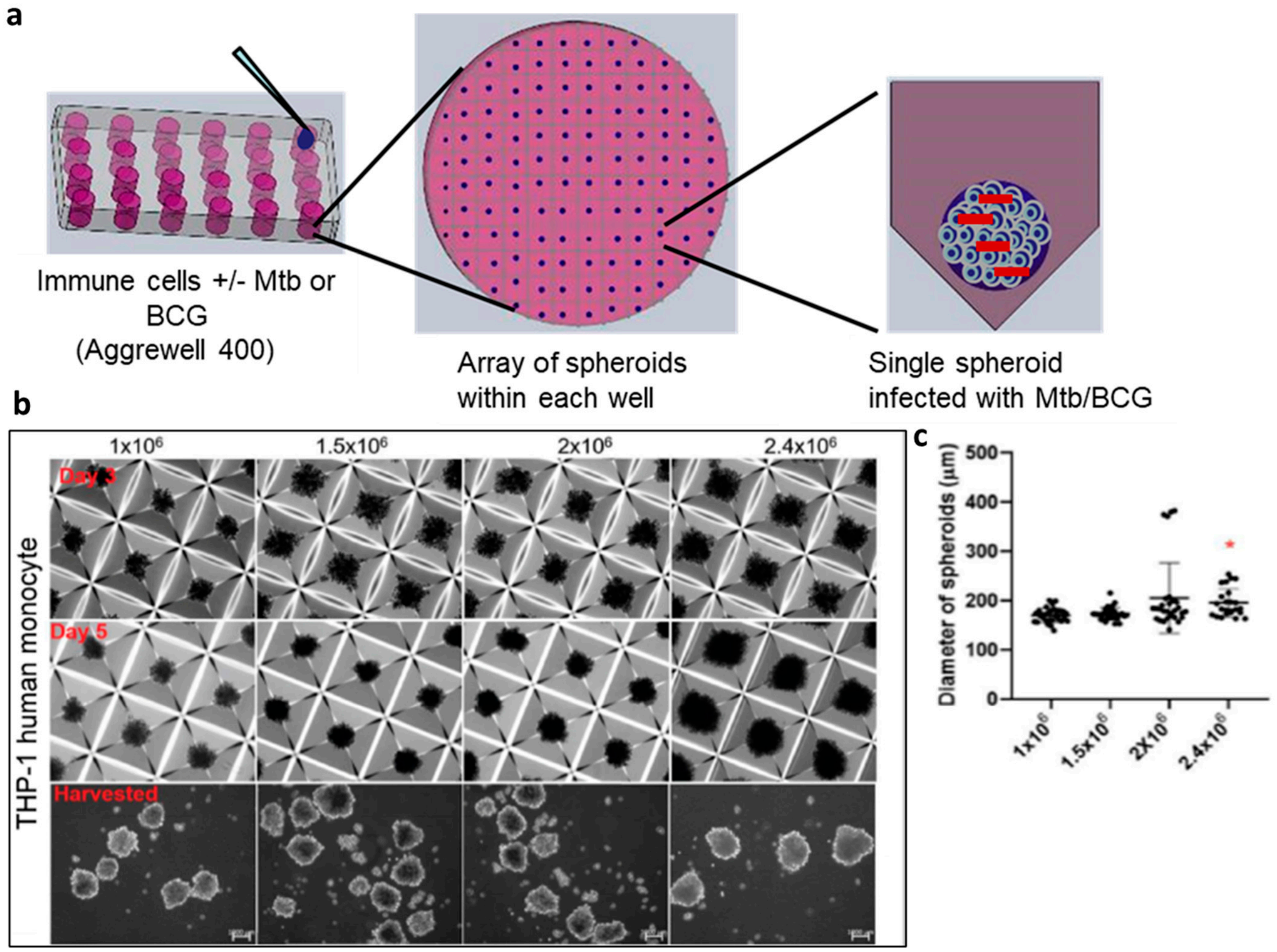

Figure S1. Formation of 3D spheroids using THP1 cells (a) schematic for formation of TB spheroid (b) generation of THP-1 spheroids using different cell numbers (c) Diameter of the spheroids estimated via Image analysis (N=24-48 spheroids/condition). * denotes statistical significance ($p<0.05$) when compared to 1x10^6 group using one-way Anova and Tukey post hoc test.

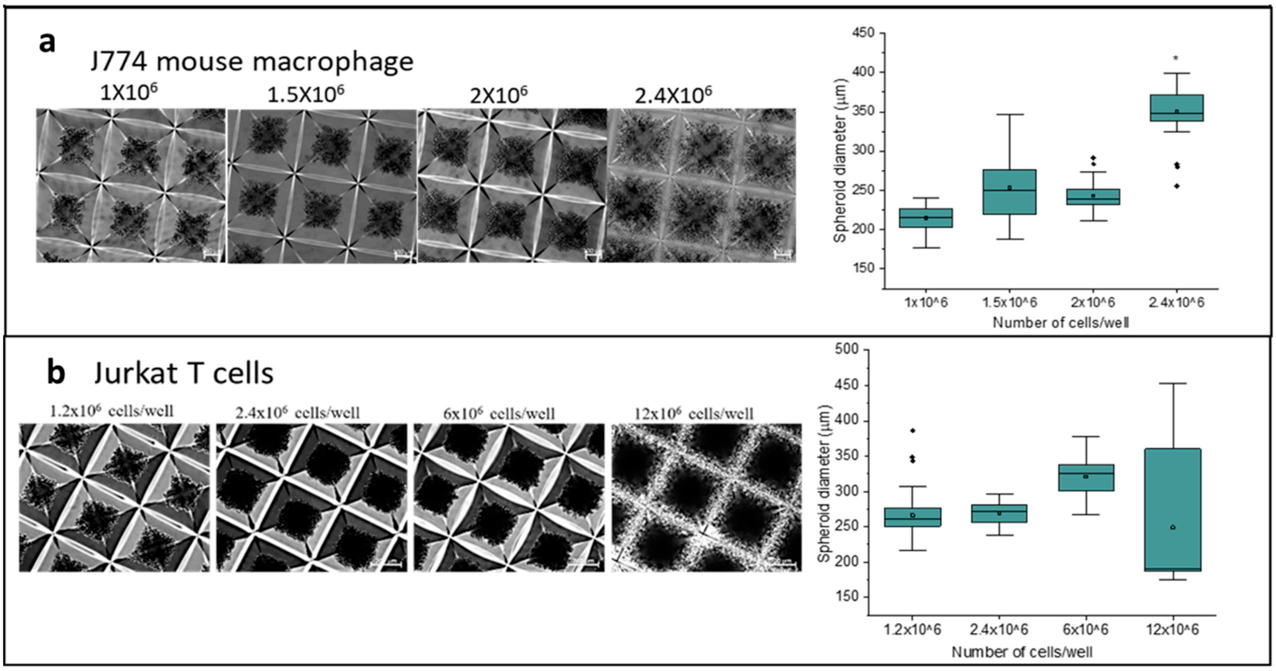

Figure S2 Validation of the model to form spheroids using other immune cells. (a) Mouse macrophage and (b) Jurkat T . Diameter of N=24-48 spheroids for each cell type are represented in the bar graphs. * denotes statistical significance ($p<0.05$) when compared to 1x10^6 group using one-way Anova and Tukey post hoc test.

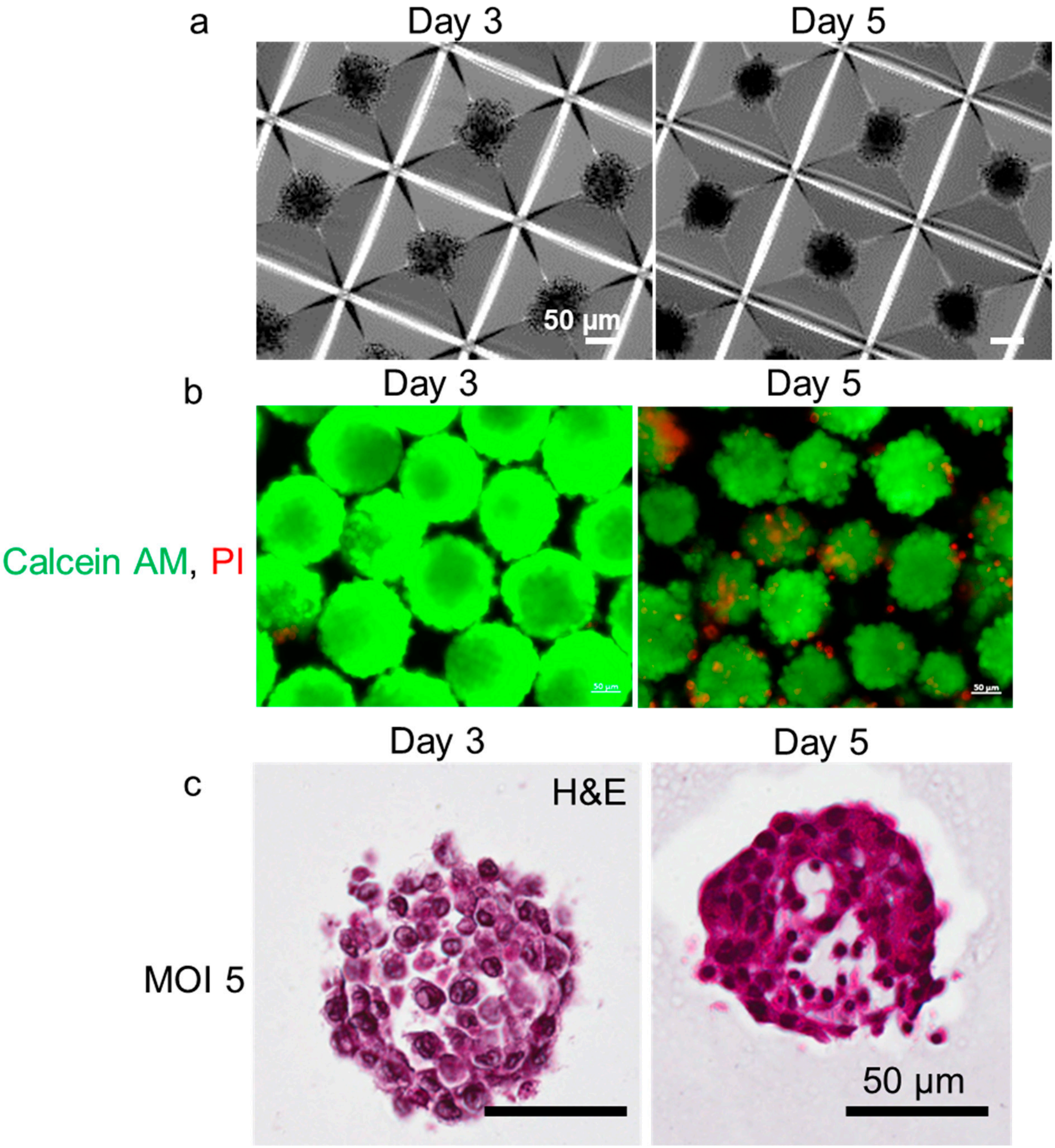

Figure S3 Time dependent tracking of spheroids (a) Brightfield images of THP-1 spheroids on day 3 and day 5 (b) Calcein AM (green-Live) and PI (red-Dead) stained spheroids on day 3 and day 5 (c)H&E-stained sections of BCG infected spheroids harvested on day 3 and 5.

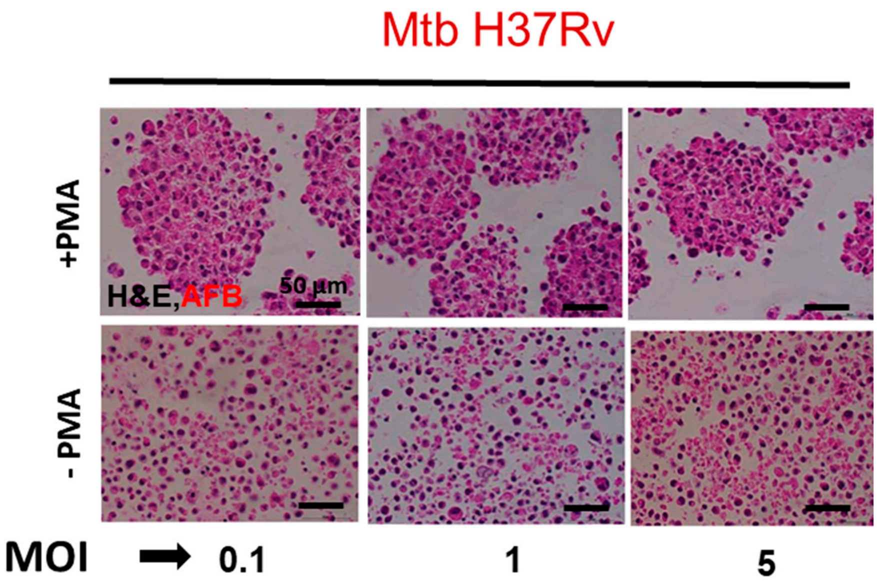

Figure S4 Histological analysis of Mtb infected spheroids. Images of 5μm sections of Mtb infected spheroid cultured with and without phorbol 12-myristate 13-acetate (PMA), stained with H&E (host cells), and acid-fast bacilli (AFB, bacteria).

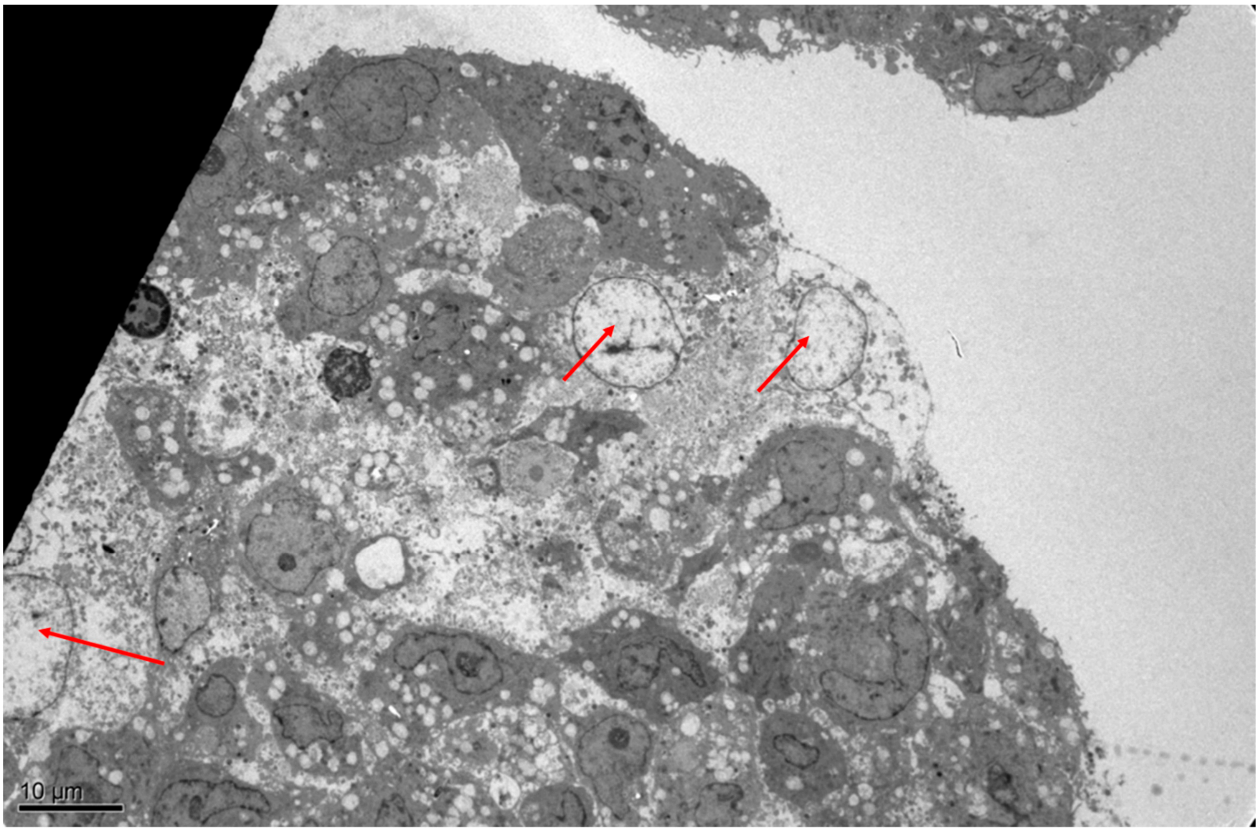

Figure S5 TEM image of TB spheroid infected with BCG mCherry. Red arrows indicate necrotic cells.

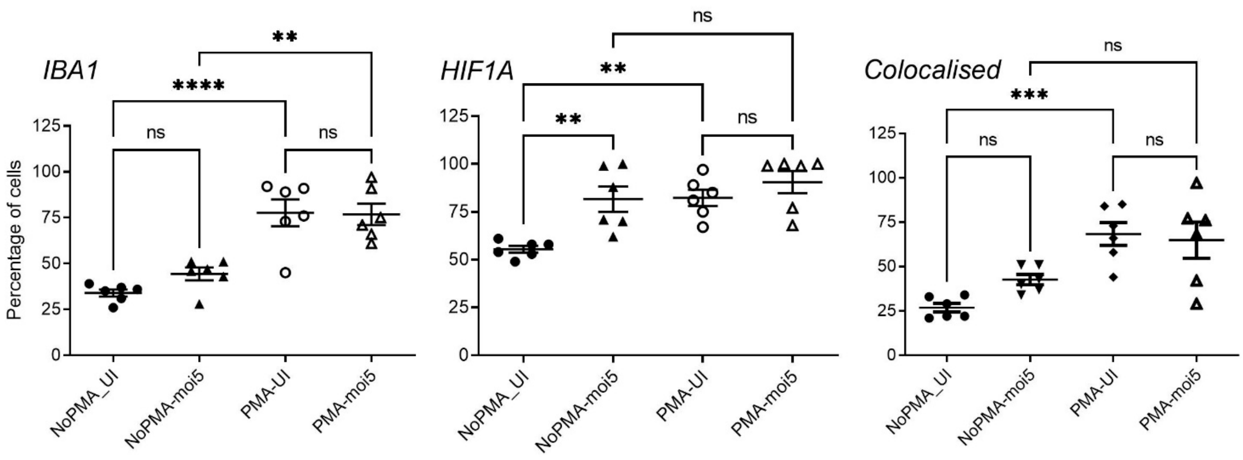

Figure S6 Quantification of co-localized IBA-1 and HIF-1α in non-activated and PMA-activated TB spheroid with or without Mtb infection. Number of cells positive for IBA-1(Green), HIF-1a (Red) or both (orange red) were manually counted from images at 63x magnification. At least 50 non-necrotic, intact cells per field were counted from each spheroid and 3-4 spheroids from each of 3 sections were counted to get the data. The data was analyzed by one-way ANOVA with Tukey's post analysis correction for multiple group comparison. *p<0.05; **p<0.001; ***p<0.0005.

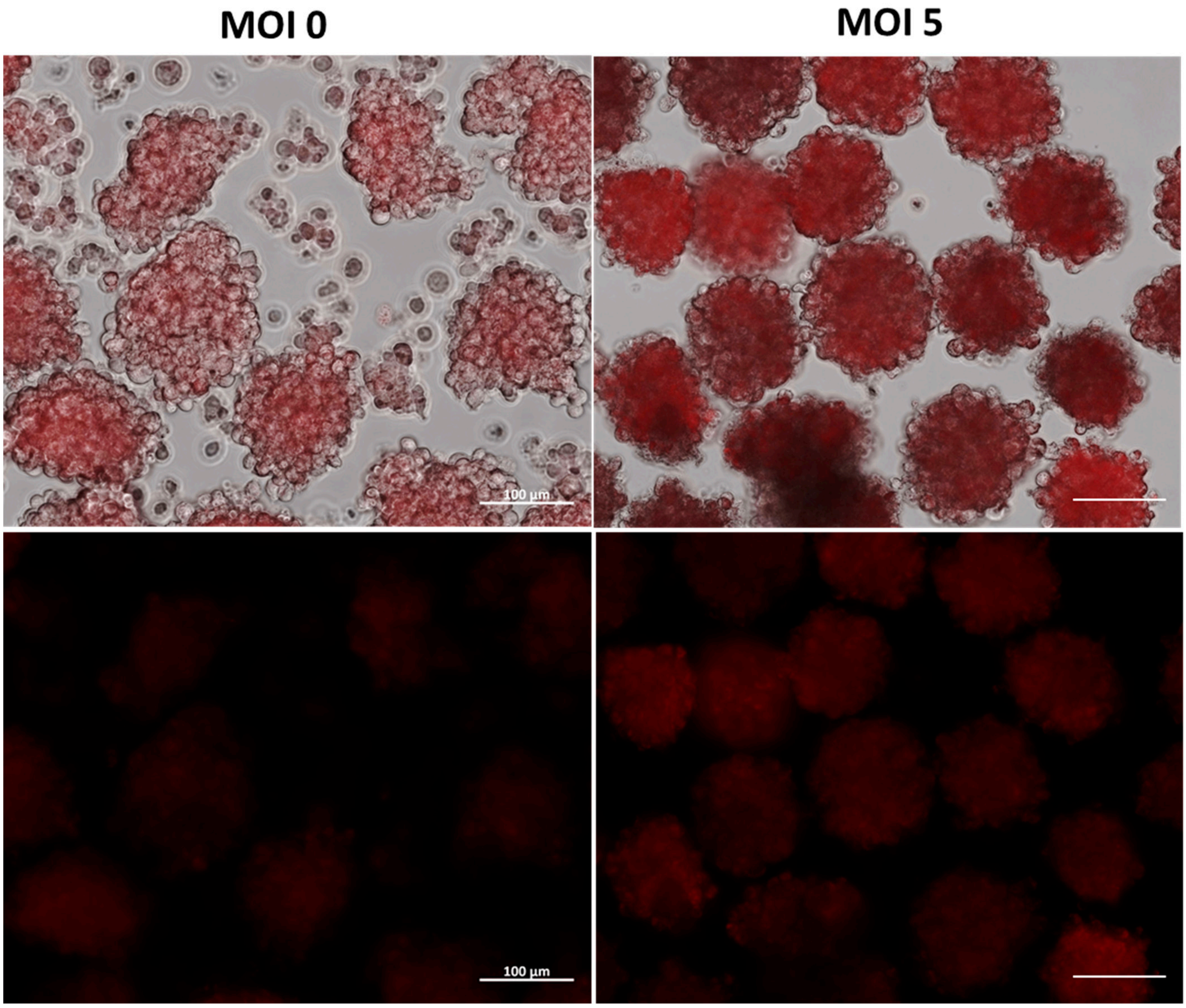

Figure S7 Nile red staining of THP-1 spheroids. Representative images of THP-1 spheroids infected with BCG mCherry (MOI 5) and no infection controls (MOI 0) stained with Nile red.

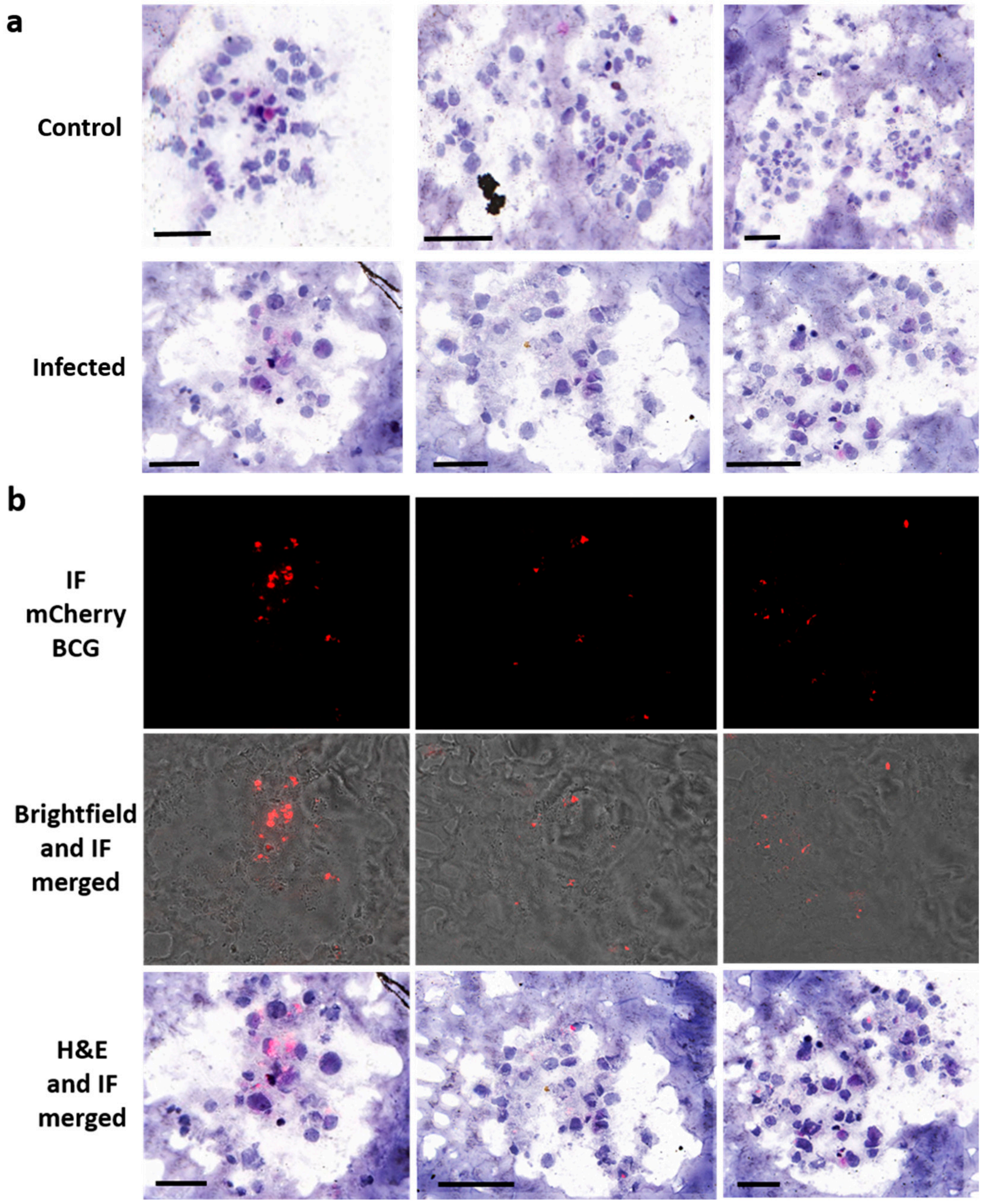

Figure S8 MALDI MSI H&E staining in replicates. (a) Hematoxylin and Eosin (H&E) stained spheroid replicates used for MSI. (b) Immunofluorescence (IF) of the mCherry BCG infected spheroid replicates (top panel). Brightfield and IF merged images of the mCherry BCG infected spheroid replicates (middle panel). H&E and IF mergend images used for MSI analysis (bottom panel). Scale bars are 100 μm.

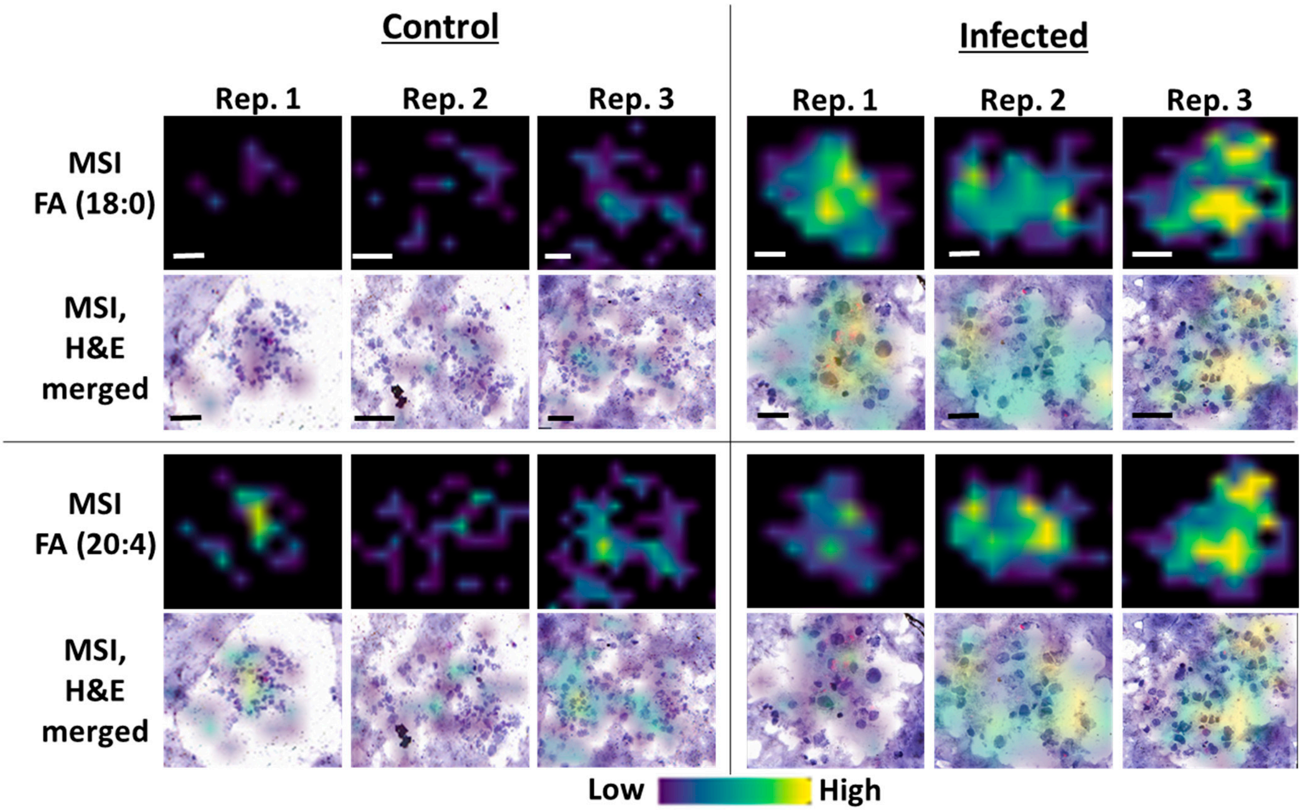

Figure S9 MSI data of fatty acids detected in control vs. infected spheroids. The overlaid MSI lipid data for the infected spheroids are with the mCherry IF-H&E merged images. Scale bars are 100 μm.

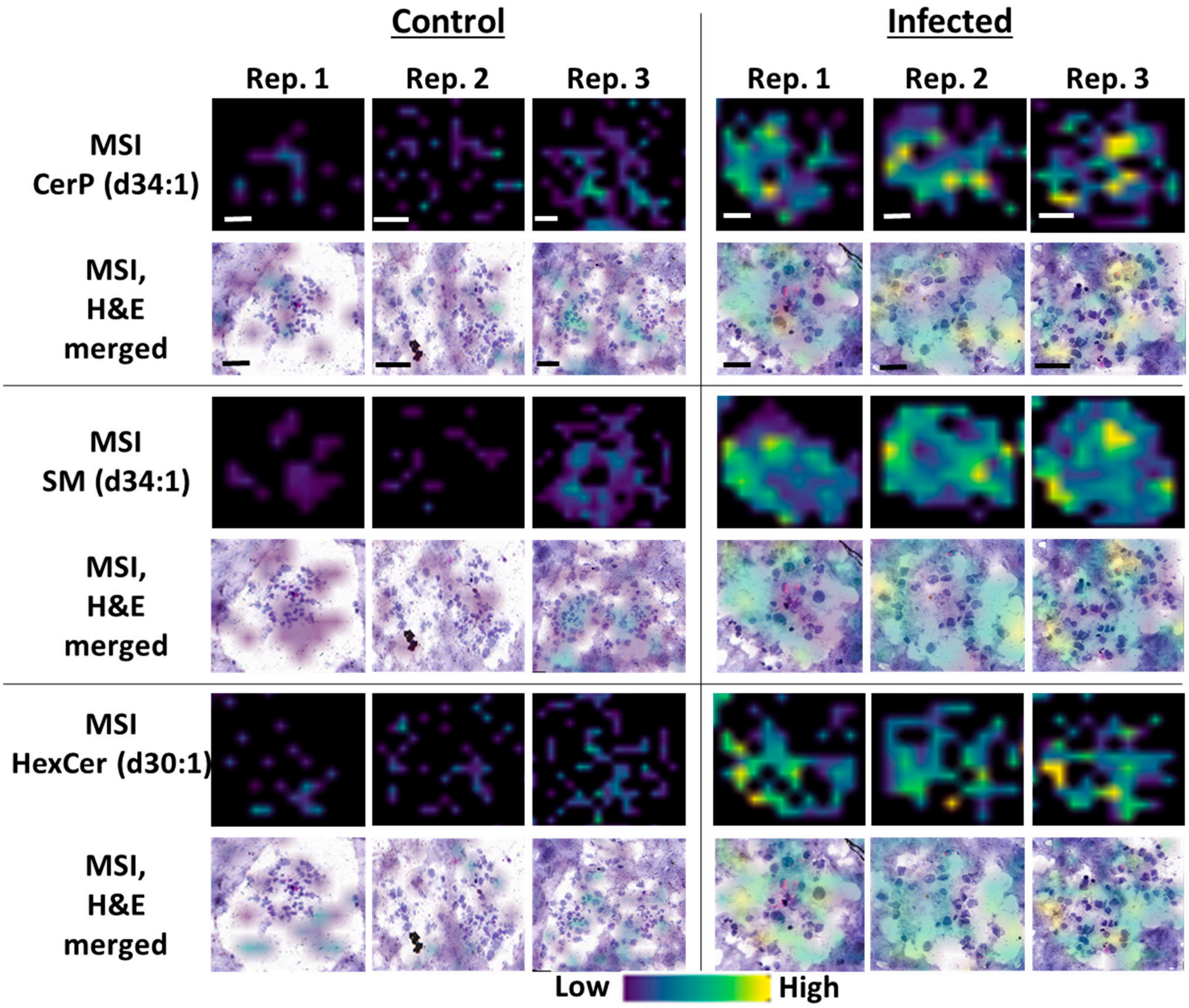

Figure S10 MSI data of sphingolipids detected in control vs. infected spheroids. The overlaid MSI lipid data for the infected spheroids are with the mCherry IF-H&E merged images. Scale bars are 100 μm.

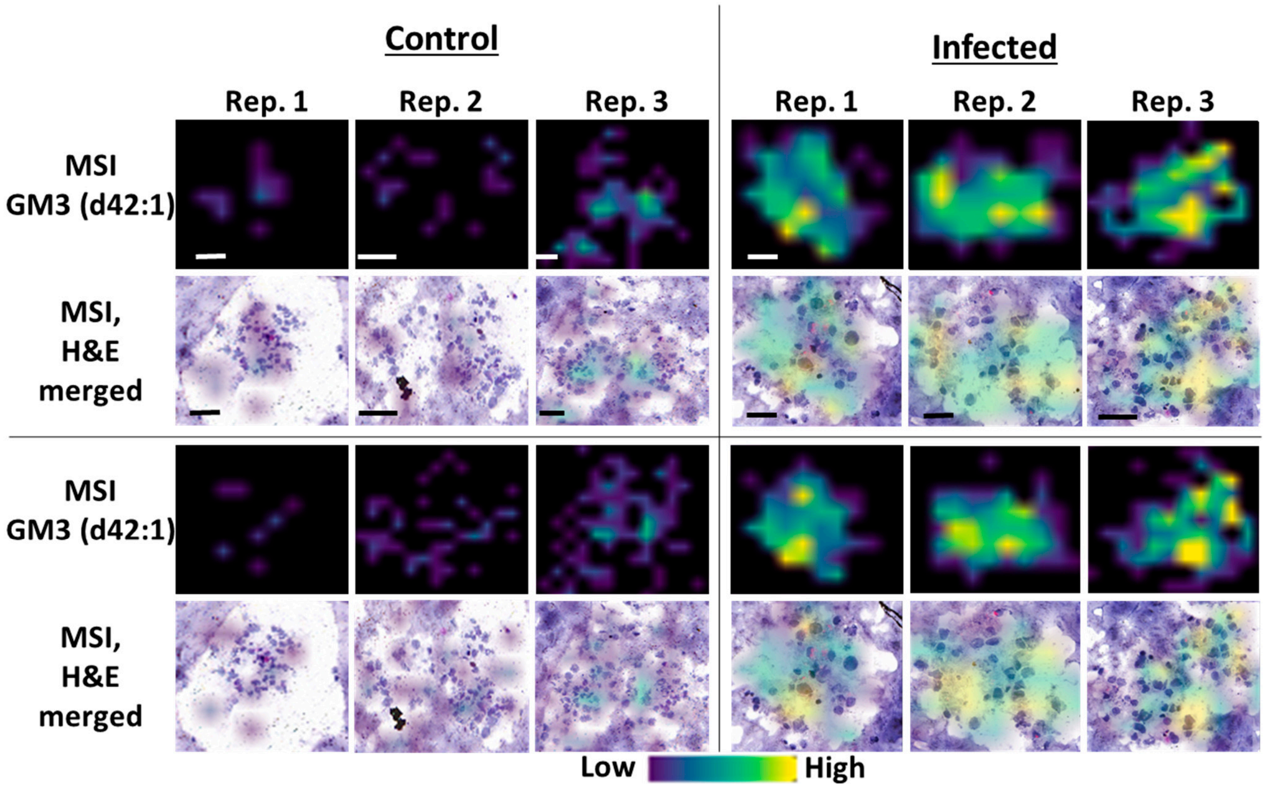

Figure S11 MSI data of gangliosides detected in control vs. infected spheroids. The overlaid MSI lipid data for the infected spheroids are with the mCherry IF-H&E merged images. Scale bars are 100 μm.

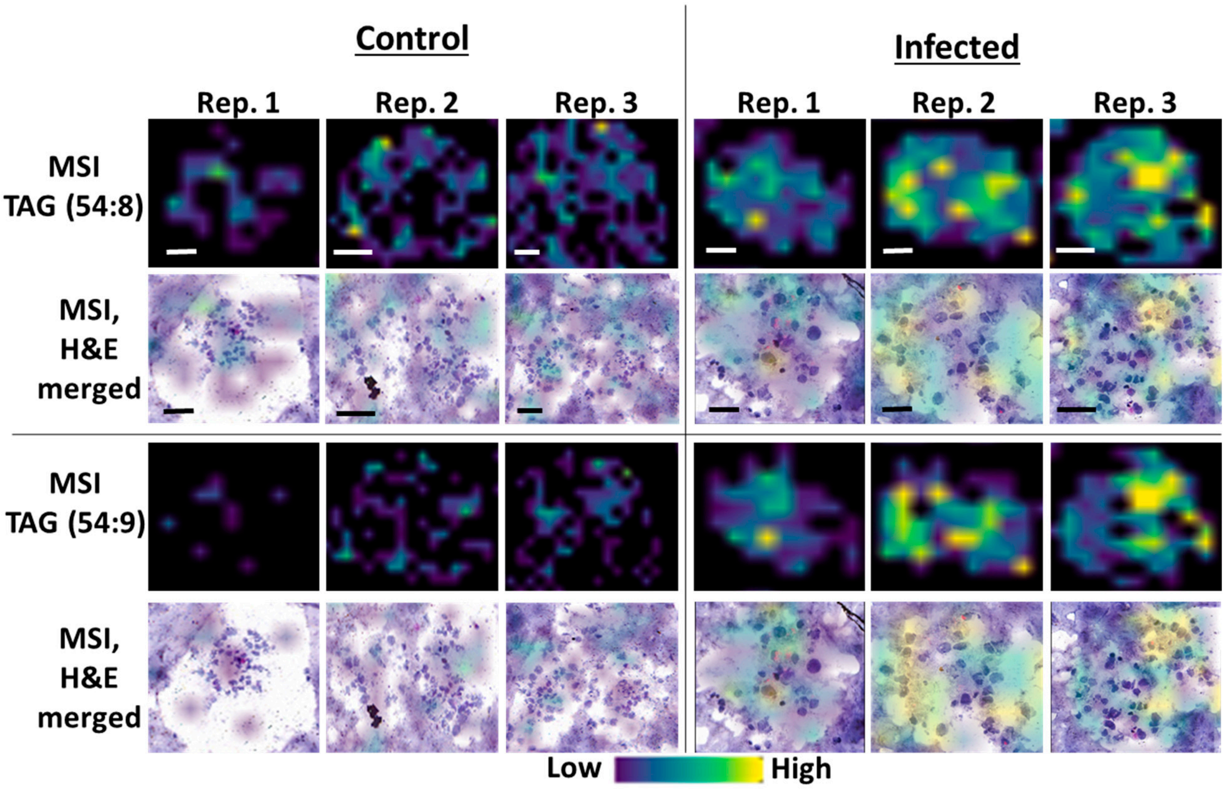

Figure S12 MSI data of triacylglycerides detected in control vs. infected spheroids. The overlaid MSI lipid data for the infected spheroids are with the mCherry IF-H&E merged images. Scale bars are 100 μm.

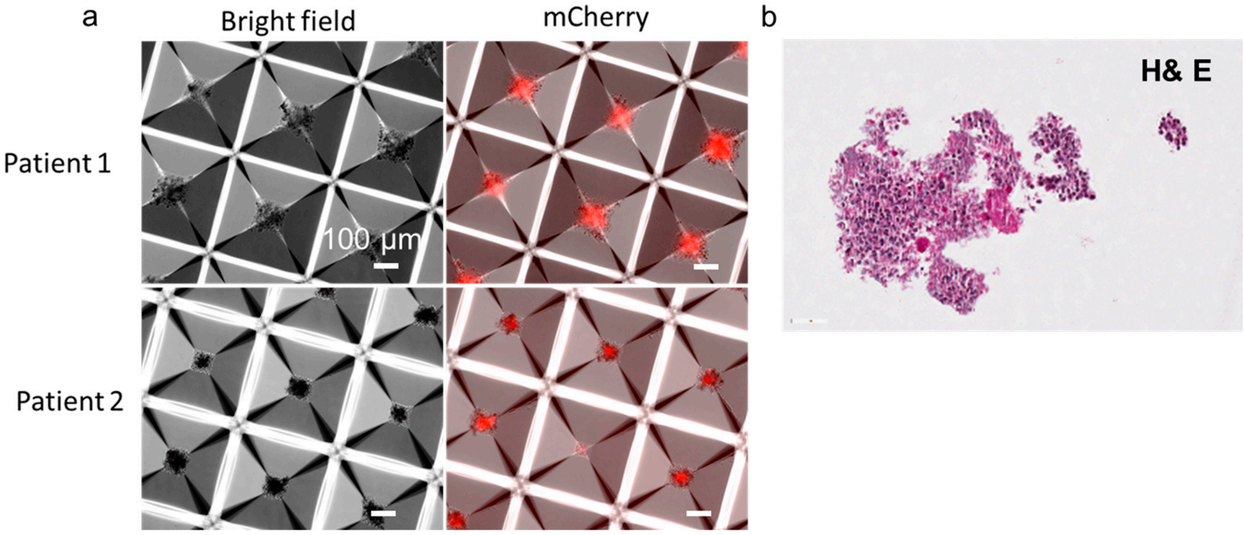

Figure S13 PBMC aggregate formation (a) Infection of PBMCs derived from two healthy donors with BCG mCherry at MOI 1 (b) H&E staining of harvested aggregate section.

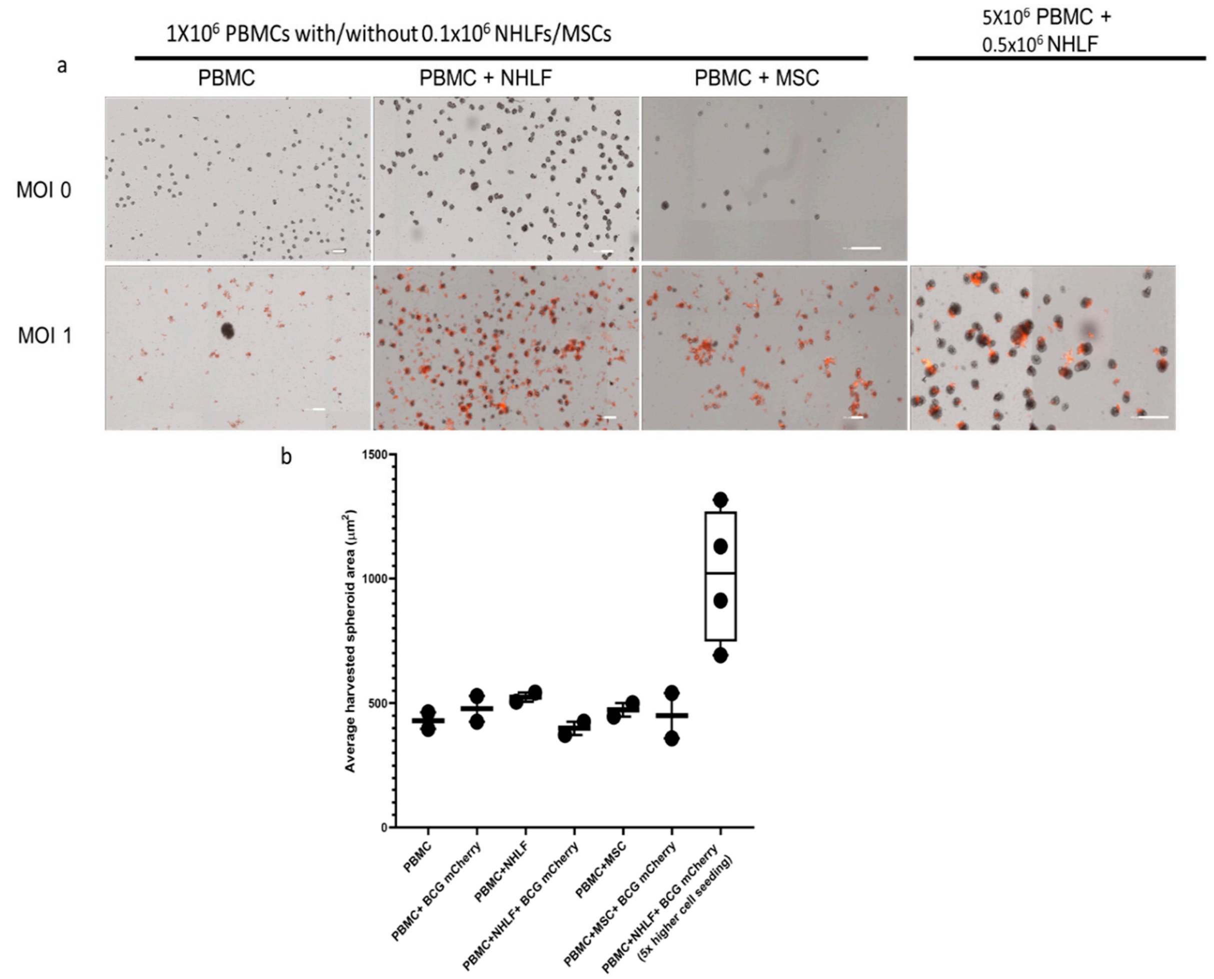

Figure S14 Formation of PBMC spheroids co-cultured with stromal cells (a)Images of PBMCs co-cultured with NHLF and MSCs. *Scale bar indicates 500 µm.* (b) Quantification of average area of harvested spheroids. N=2 wells/condition for all the groups except PBMC+ NHLF+ BCG mCherry (5x higher seeding), where N=4 wells/condition were used and the average for each group was calculated based on ~1200 spheroids/well.
